# Derivatives of Differentiation-Inducing Factor 1 Differentially Control Chemotaxis and Stalk Cell Differentiation in *Dictyostelium discoideum*

**DOI:** 10.3390/biology12060873

**Published:** 2023-06-16

**Authors:** Hidekazu Kuwayama, Haruhisa Kikuchi, Yuzuru Kubohara

**Affiliations:** 1 Graduate School of Life and Environmental Sciences, University of Tsukuba, Tsukuba 305-8572, Japan; kuwayama.hidekazu.fu@u.tsukuba.ac.jp; 2 Division of Natural Medicines, Faculty of Pharmacy, Keio University, Tokyo 105-8512, Japan; halkiku@keio.jp; 3 Laboratory of Health and Life Science, Graduate School of Health and Sports Science, Juntendo University, Inzai 270-1695, Japan

**Keywords:** *Dictyostelium*, DIF, GbpB, RegA, RdeA, DhkC, stalk cell differentiation, chemotaxis

## Abstract

**Simple Summary:**

In this study, we examined the effects of nine DIF derivatives on chemotactic cell movement toward cAMP and compared their chemotaxis-modulating activity and stalk cell differentiation–inducing activity in wild-type and mutant strains in *Dictyostelium discoideum*. We found that the DIF derivatives differentially affected chemotaxis and stalk cell differentiation, suggesting that DIF-1 and DIF-2 have at least three receptors: one for stalk cell induction and two for chemotaxis modulation.

**Abstract:**

Differentiation-inducing factors 1 and 2 (DIF-1 and DIF-2) are small lipophilic signal molecules that induce stalk cell differentiation but differentially modulate chemotaxis toward cAMP in the cellular slime mold *Dictyostelium discoideum*; DIF-1 suppresses chemotactic cell movement in shallow cAMP gradients, whereas DIF-2 promotes it. The receptor(s) for DIF-1 and DIF-2 have not yet been identified. We examined the effects of nine derivatives of DIF-1 on chemotactic cell movement toward cAMP and compared their chemotaxis-modulating activity and stalk cell differentiation–inducing activity in wild-type and mutant strains. The DIF derivatives differentially affected chemotaxis and stalk cell differentiation; for example, TM-DIF-1 suppressed chemotaxis and showed poor stalk-inducing activity, DIF-1(3M) suppressed chemotaxis and showed strong stalk-inducing activity, and TH-DIF-1 promoted chemotaxis. These results suggest that DIF-1 and DIF-2 have at least three receptors: one for stalk cell induction and two for chemotaxis modulation. In addition, our results show that the DIF derivatives can be used to analyze the DIF-signaling pathways in *D. discoideum*.

## 1. Introduction

The vegetative amoebae of the cellular slime mold *Dictyostelium discoideum* grow by eating bacteria. Upon starvation, the cells gather to form a slug-shaped multicellular aggregate and differentiate into two distinct cell types: prespore and prestalk cells. Eventually, the cells form a fruiting body consisting of spores and a multicellular stalk. During the morphogenesis, extracellular cAMP is essential for cell differentiation, and it also acts as a chemoattractant that induces the cells to gather to form a multicellular aggregate [1,2,3]. Thus, *D. discoideum* is an excellent model organism for the study of both cell differentiation and chemotaxis.

**Figure 1:**
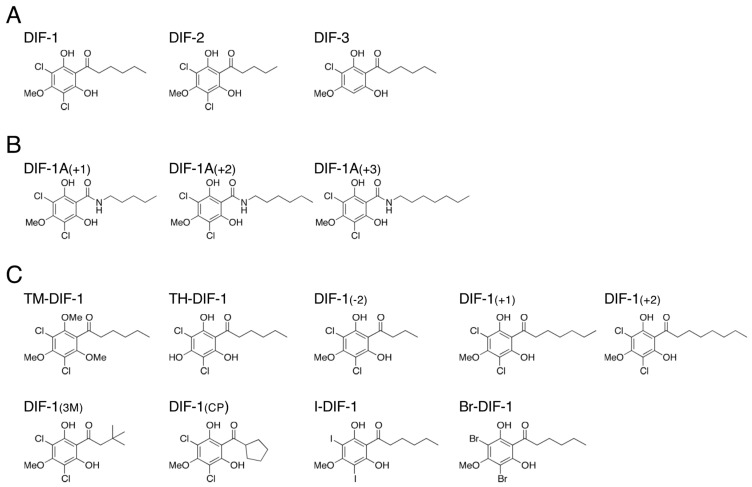
Chemical structure of (**A**) DIFs 1–3, (**B**) amide derivatives of DIF-1, and (**C**) the nine DIF derivatives used in this study. Note that DIF-1A(+1), DIF-1A(+2), and DIF-1A(+3) were referred to as DIF-1[A+1], DIF-1[A+2], and DIF-1[A+3] under our previous nomenclature [11].

Differentiation-inducing factors 1, 2, and 3 (DIF-1, DIF-2, and DIF-3) (Figure 1A) are characteristic chlorinated alkylphenones that were originally identified as stalk cell differentiation–inducing factors in *D. discoideum* [4,5,6,7]. DIF-1 is most active in inducing stalk cell formation in vitro, DIF-2 possesses ~40% of the specific activity of DIF-1, and DIF-3, which is a degradation product of DIF-1, possesses only ~5% of the specific activity of DIF-1 [6,7,8,9]. We previously reported that DIF-1 and DIF-2 can also function as negative and positive modulators of chemotaxis, respectively, in shallow cAMP gradients, but that DIF-3 does not affect chemotaxis [10]. However, no receptor(s) for DIF-1 and DIF-2 have been identified to date.

**Figure 2:**
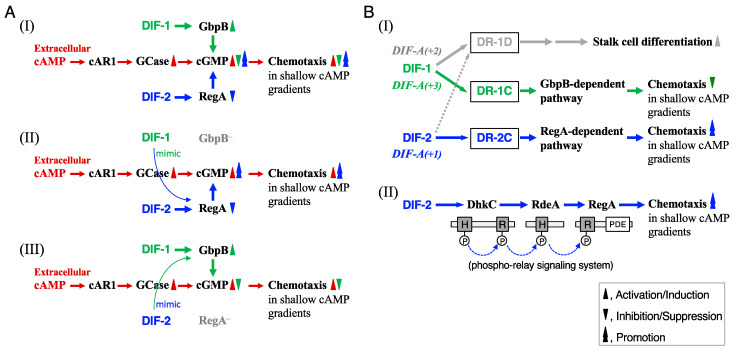
(**A**) Proposed scheme for the actions of DIF-1 and DIF-2 in modulating chemotaxis [10]. (**I**) *Dictyostelium* cells show chemotactic movement toward extracellular cAMP, which induces chemotaxis by binding to the cell surface cAMP receptor (cAR1), followed by activation of guanylyl cyclase (GCase) and an increase in intracellular cGMP. In shallow cAMP gradients, DIF-1 inhibits chemotaxis toward cAMP, at least in part, via activation of the cGMP-PDE GbpB and a subsequent decrease in intracellular cGMP, whereas DIF-2 enhances chemotaxis, at least in part, via a RegA (a cAMP-PDE)-dependent pathway and a subsequent increase in intracellular cGMP. However, at high concentrations of DIFs (e.g., 100 nM), cross-talk can occur, and DIF-1 and DIF-2 both enhance chemotaxis in *gbpB*^−^ cells (**II**) and inhibit chemotaxis in *regA*^−^ cells (**III**). (**B**) (**I**) Proposed scheme for the actions of DIF compounds in inducing stalk cell differentiation and modulating chemotaxis via three putative DIF receptors [11,20,21]. During normal development, DIF-1 would induce stalk cell differentiation, at least in part, via a DIF receptor (DR-1D) and negatively modulate chemotaxis via another DIF receptor (DR-1C) and a GbpB-dependent pathway. In contrast, DIF-2 would function mainly as a positive modulator for chemotaxis, at least in part, via another DIF receptor (DR-2C) and a RegA-dependent pathway. The artificial compounds, DIF-1A(+2) and DIF-1A(+3), would be efficient stalk cell inducers and chemotaxis modulators, possibly via DR-1D and DR-1C, respectively. DIF-1A(+1), like DIF-2, would induce stalk cell differentiation via DR-1D and modulate chemotaxis via DR-2C. Note that the DIF receptors that were DR-1, DR-2 and DR-3 under our previous nomenclature [11] are referred to as DR-1D, DR-1C, and DR-2C, respectively, in our previous [21] and present study in order to match the names of the receptors and their ligands, DIF-1 and DIF-2. (**II**) Proposed scheme for the actions of DIF-2 via the DhkC–RdeA–RegA phospho-relay pathway. The schematic diagram of the phospho-relay pathway illustrates the previously proposed model [22,23,24,25]; DhkC, *Dictyostelium* histidine kinase C, phosphorylates itself and passes the phosphate through the relay by RdeA to RegA, resulting in activation of RegA (cAMP phosphodiesterase). DIF-2 modulates chemotaxis, at least in part, via the *Dictyostelium* phospho-relay signaling system, DhkC–RdeA–RegA pathway [20]. H, a site of histidine phosphorylation. R, receiver domain. PDE, phosphodiesterase. The catalytic domain of DhkC is omitted for simplicity.

DIF-1 and DIF-2 induce stalk cell differentiation, at least in part, via increases in cytosolic calcium and proton concentrations [12,13,14]. We have shown, with the mutant strains lacking a cGMP-phosphodiesterase (PDE), GbpB [15,16,17], and a cAMP-PDE, RegA [18], that DIF-1 suppresses chemotaxis, at least in part, via a GbpB-dependent pathway, whereas DIF-2 promotes chemotaxis, at least in part, via a RegA-dependent pathway (Figure 2AI) [10]. The results of our experiments using DIFs and chemically synthesized amide derivatives of DIF-1 (Figure 1B) [19] suggested that there might be at least three receptors for DIF-1 and DIF-2: DIF-1 receptor responsible for stalk cell differentiation (DR-1D), DIF-1 receptor responsible for chemotaxis modulation (DR-1C), and DIF-2 receptor responsible for chemotaxis modulation (DR-2C) (Figure 2BI) [11]. Some of the amide derivatives of DIF-1 have activities similar to DIF-1 and DIF-2. DIF-1A(+1) promotes chemotaxis similar to DIF-2, DIF-1A(+2) induces stalk cell differentiation similar to DIF-1, and DIF-1A(+3) suppresses chemotaxis similar to DIF-1 (Figure 2BI) [11]. These amide derivatives could be useful tools for identifying and characterizing the DIF receptors.

We previously assessed the chemical structure–activity relationship of DIF derivatives (some of which are shown in Figure 1C) on cell differentiation; we showed that some of the derivatives can induce stalk cell differentiation in cells of a DIF-deficient strain HM44 [26]. However, the effects of these DIF derivatives on chemotaxis have not been examined.

In this study, to further assess the structure–activity relationships of DIF derivatives and characterize the DIF receptors, we examined the effects of nine DIF derivatives (Figure 1C) on both chemotaxis and stalk cell differentiation using chemotaxis-related and differentiation-related mutant strains. We show here that the DIF derivatives differentially control chemotaxis and stalk cell differentiation in vitro and suggest that at least three DIF-1 and DIF-2 receptors control chemotaxis and cell differentiation in *D. discoideum.*

## 2. Materials and Methods

### 2.1. Strains and Reagents

*Dictyostelium discoideum* wild type Ax2, DIF-deficient HM1030 (*dmtA*^−^) [27], *gbpB*^−^ [15,16,17], *regA*^−^ [18,28], *rdeA*^−^ [22], and *dhkC*^−^ [20] cells were used. DIF-1, DIF-2, DIF-3, and DIF-1 derivatives were synthesized as previously described [19,26]; they were dissolved in dimethylsulfoxide (DMSO) and stored at −20 °C. A hydrophobic index (cLogP) of each derivative was calculated by the use of the ChemDraw Professional 20.0 software (PerkinElmer Informatics, Waltham, MA, USA).

### 2.2. Assay for In Vitro Stalk Cell Differentiation

Cells were grown in an in vitro monolayer culture for stalk cell induction as described previously [11,29]. HM1030 cells were grown at 21 °C for about 2 days in association with *Klebsiella aerogenes* on a modified SM agar plate [30]. The cells were harvested and washed with a salt solution (10 mM NaCl, 10 mM KCl) several times to remove bacteria, and then allowed to differentiate at 21 °C in 3.5 cm tissue culture dishes (5–10 × 10^5^ cells/dish); each dish contained 1.2 mL of stalk medium (10 mM Mes-KOH pH 6.2, 2 mM NaCl, 10 mM KCl, 1 mM CaCl_2_, 50 μg/mL penicillin, 100 μg/mL streptomycin sulfate). At 8 h, 10 μL of 0.5 M cAMP was added (to a final concentration of ~4.2 mM). At 24 h, cAMP was removed by washing the cells three times with 1 mL of stalk medium. Cells were further incubated for 24 h (total incubation time, 48 h) in 1.2 mL of stalk medium containing 10–20 nM of DIF compound or 0.1–0.2% (*v/v*) DMSO (control). Cells were observed by phase-contrast microscopy to determine stalk cell differentiation; usually more than 150 cells/dish were scored.

### 2.3. Assay for Chemotaxis

The chemotaxis assay (i.e., the small-population assay) was performed as previously described [10]. Cells were cultured at 21 °C in HL5 medium containing 100 units/mL benzylpenicillin potassium and 100 μg/mL streptomycin sulfate, as previously described [10]. Cells were harvested by centrifugation (350× *g* for 2 min), washed in phosphate buffer (PB) (10 mM Na_2_HPO_4_/KH_2_PO_4_, pH 6.5), and starved at a density of 1 × 10^7^ cells/mL in PB buffer for 1 h. Over a period of 5 h, cAMP was added in a pulsatile fashion every 6 min to a final concentration of 30 nM. The starved cells were washed twice with PB, and triplicate samples were suspended in PB containing a DIF derivative to a final concentration of 5 × 10^6^ cells/mL. For each sample, ten droplets of starved cells (<0.2 μL) were placed on a 10 cm plate containing 10 mL of non-nutrient hydrophobic agar (0.7% hydrophobic agar containing 10 mM Na_2_HPO_4_/KH_2_PO_4_, pH 6.5, and 3 mM caffeine) with 0.1% DMSO (vehicle) or a DIF compound (10 nM or 100 nM). Chemotaxis toward cAMP was tested after 30 min by placing a second 0.1-μL droplet, with the indicated concentration (10^−10^–10^−7^ M) of cAMP, next to the droplet of cells. Then, the distribution of the cells in the droplet was observed, and the droplet was scored as positive when at least twice as many cells were pressed against the side of the droplet closer to the higher cAMP concentration than against the other side of the droplet. The percentage of positive droplets was assessed, and data for each set of triplicate agar plates are presented as the mean and standard deviation (SD).

### 2.4. Statistical Analysis

Statistical analysis was performed by using Student’s *t*-test (two-tailed, unpaired), and values of *p* < 0.05 were considered statistically significant.

## 3. Results

### 3.1. Effects of DIFs (100 nM) on Chemotaxis in Ax2, gbpB^−^, and regA^−^ Cells

**Figure 3:**
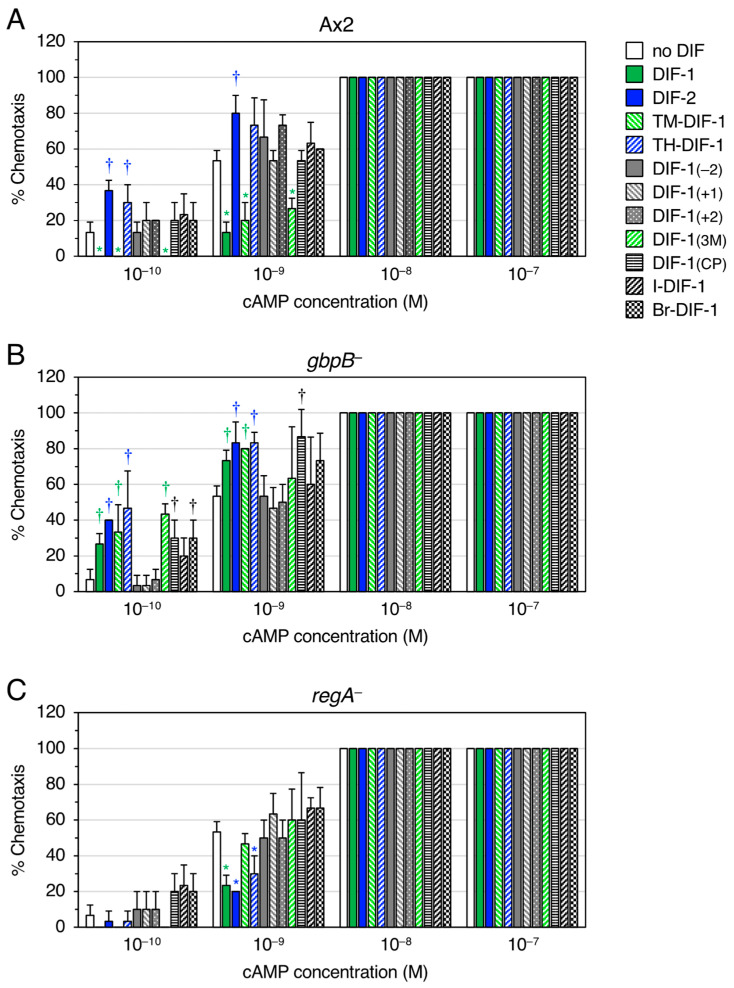
Effect of DIFs (100 nM) on chemotaxis in Ax2, *gbpB^−^*, and *regA^−^* cells. (**A**) Ax2, (**B**) *gbpB^−^*, and (**C**) *regA^−^* cells were starved for 6 h in shake-culture, and cell droplets were spotted on PB agar containing 3 mM caffeine (Control) plus 100 nM DIF compounds. Cells were assayed for chemotaxis toward the indicated doses of cAMP; 10 cell droplets were examined for each cAMP concentration. Data are presented as the mean ± SD of triplicate measurements (*n* = 3) for one experiment. * and † signify statistically significant inhibition and promotion of chemotaxis, respectively; *p* < 0.05 versus Control.

We examined the effects of DIF-1, DIF-2, and nine DIF derivatives (Figure 1C) on chemotaxis toward various concentrations of cAMP in Ax2 cells (Figure 3A). In shallow gradients of cAMP, DIF-1 at 100 nM significantly suppressed chemotaxis, and DIF-2 at 100 nM significantly promoted chemotaxis as reported previously [10]. Interestingly, TM-DIF-1 and DIF-1(3M) significantly suppressed chemotaxis, like DIF-1, whereas TH-DIF-1 promoted it, like DIF-2. The other six DIF derivatives showed no significant effects on chemotaxis.

We have previously shown that DIF-1 suppresses chemotaxis via a GbpB-dependent pathway and DIF-2 promotes it via a RegA-dependent pathway (Figure 2AI) [10]. To assess if the actions of the DIF derivatives also require these PDEs, we examined and compared the effects of DIF compounds (100 nM) on chemotaxis in *gbpB*^−^ and *regA*^−^ cells (Figure 3B,C). As hypothesized on the basis of Figure 2AII, when TM-DIF-1 and DIF-1(3M) were administered to *gbpB*^−^ cells, their original activity, like that of DIF-1, was abolished, and they mimicked the activity of DIF-2, promoting chemotaxis (Figure 3B). Additionally, as hypothesized on the basis of Figure 2AIII, when TH-DIF-1 was administered to *regA*^−^ cells, its original activity, like that of DIF-2, was abolished, and it mimicked the activity of DIF-1, suppressing chemotaxis (Figure 3C).

### 3.2. Effects of DIFs (10 nM) on Chemotaxis in Ax2, regA^−^, rdeA^−^, and dhkC^−^ Cells

**Figure 4:**
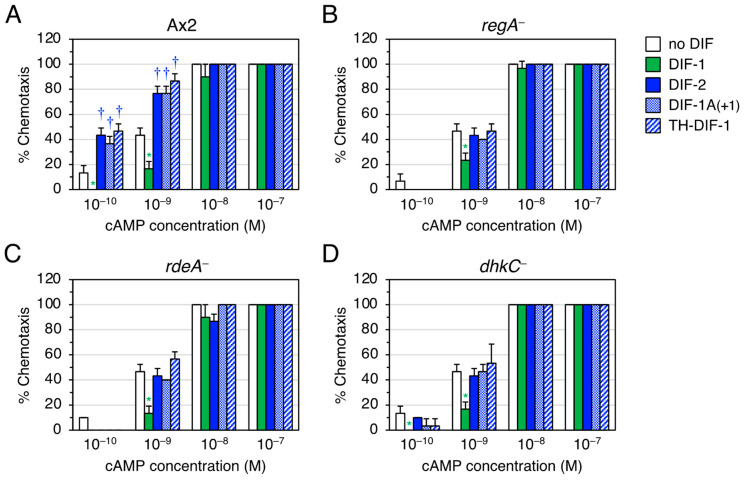
Effect of DIFs (10 nM) on chemotaxis in Ax2, *regA^−^*, *rdeA^−^*, and *dhkC^−^* cells. (**A**) Ax2, (**B**) *regA^−^*, (**C**) *rdeA^−^*, and (**D**) *dhkC^−^* cells were starved for 6 h in shake-culture, and cell droplets were spotted on PB agar containing 3 mM caffeine (Control) plus 10 nM DIF compounds. Cells were assayed for chemotaxis toward the indicated doses of cAMP; 10 cell droplets were examined for each cAMP concentration. Data are presented as the mean ± SD of triplicate measurements (*n* = 3) for one experiment. * and † signify statistically significant inhibition and promotion of chemotaxis, respectively; *p* < 0.05 versus Control.

We then focused on the function of DIF-2-type derivatives involving RegA. RegA, a phosphodiesterase [18,28], is a component of the *Dictyostelium* phospho-relay signal transduction system, DhkC–RdeA–RegA pathway [22,23,24,25]; DhkC is a *Dictyostelium* histidine kinase [25], and rapid development A (RdeA) is a member of the histidine-containing phosphotransfer proteins (phosphotransferases) that participate in multistep phosphoryl relays [22]. DIF-2 functions through the DhkC–RdeA–RegA pathway (Figure 2BII) [20]. To assess if the DIF-2-type derivatives also function via the DhkC–RdeA–RegA pathway, we examined the effects of TH-DIF-1 and DIF-1A(+1) on chemotaxis toward 10^−10^–10^−7^ M cAMP in Ax2, *regA^−^*, *rdeA^−^*, and *dhkC^−^* cells (Figure 4). As expected, DIF-2, TH-DIF-1, and DIF-1A(+1) at 10 nM significantly promoted chemotaxis in shallow cAMP gradients in Ax2 cells (Figure 4A), but not in *regA^−^*, *rdeA^−^*, or *dhkC^−^* cells (Figure 4B–D). These results suggest that TH-DIF-1 and DIF-1A(+1) function via the DhkC–RdeA–RegA signaling pathway.

### 3.3. Effects of DIFs on Stalk Cell Formation in HM1030 Cells

**Figure 5:**
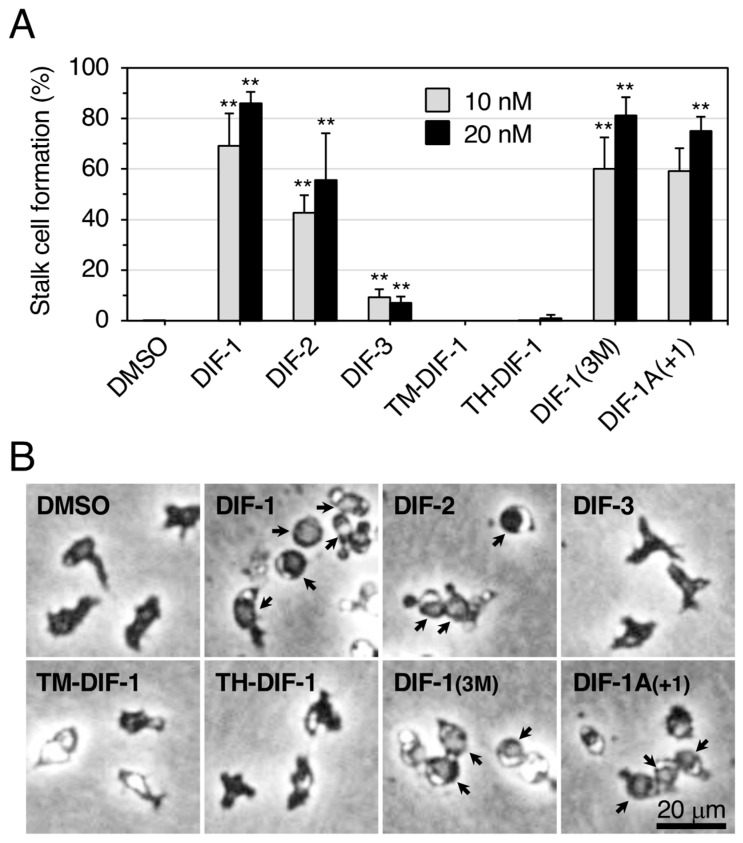
Effect of DIFs on stalk cell differentiation in HM1030 cells. (**A**) Cells were incubated without additives for 8 h, with ~4.2 mM cAMP for 16 h, and then with 0.1% or 0.2% DMSO (vehicle), or 10 nM or 20 nM DIF compounds for 24 h (total 48 h), and the stalk cells (% of total cells) were counted by using phase-contrast microscopy. Data are presented as the mean ± SD (bars) of three independent experiments (*n* = 3). ** *p* < 0.01 versus DMSO control. (**B**) Representative photos of cells after treatment with 0.1% DMSO or the indicated DIF compounds at 10 nM. Arrows indicate stalk cells.

We compared the effects of seven DIF compounds on stalk cell formation in an in vitro monolayer culture of HM1030 cells (Figure 5), an Ax2-derived DIF-deficient strain that lacks des-methyl-DIF-1 methyl transferase (an enzyme required for conversion of TH-DIF-1 to DIF-1 during DIF-1 biosynthesis [31]. HM1030 cells cannot differentiate into stalk cells in vitro unless supplied with DIF-1 [11,27,32]. DIFs 1–3 at 10 or 20 nM induced stalk cell formation as expected in HM1030 cells. TM-DIF-1 and TH-DIF-1 scarcely induced stalk cell formation, whereas DIF-1(3M) and DIF-1A(+1) induced it to the same extent as DIF-1. These results for HM1030 cells are largely consistent with our previous observations for HM44 cells [19,26], another DIF-deficient strain, which is derived from the V12M2 strain [33] (Table 1). However, the results for TH-DIF-1 were notable; it strongly induced stalk cell formation in HM44 cells but not in HM1030 cells (Table 1). We attribute this result to the following reasons: since TH-DIF-1 is the immediate precursor of DIF-1 during DIF-1 biosynthesis [31], TH-DIF-1 could have been converted to DIF-1 by des-methyl-DIF-1 methyl transferase and thus induced stalk cell formation in HM44 cells. Therefore, in HM44 cells, TH-DIF-1 itself did not function as a stalk cell inducer, its derivative did.

## 4. Discussion

### 4.1. Toward Identification of Receptors for DIF-1 and DIF-2

Although DIF-1 and DIF-2 play important roles in cell differentiation and chemotaxis of *D. discoideum* (Figure 2), 35 years after their discovery [4,5], their receptors have not yet been identified. Insall and Kay [34] identified a specific DIF-binding protein from *Dictyostelium* cell lysates by using tritiated DIF-1, but since then, complete identification of the DIF receptor has not been achieved.

**Figure 6:**
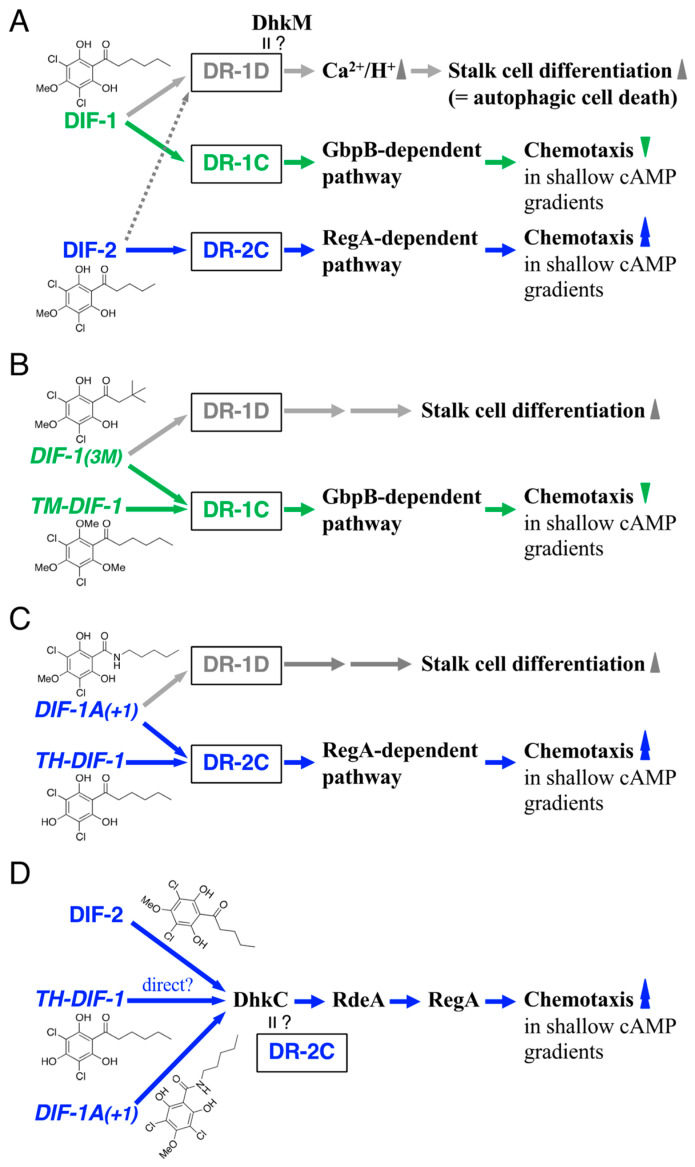
(**A**) Chemical structures of DIF-1 and DIF-2 and scheme for their actions in inducing stalk cell differentiation and modulating chemotaxis. We assume here that DIF-1 would induce stalk cell differentiation via its receptor DR-1D and subsequent increases in cytoplasmic Ca^2+^ and H^+^ concentrations, at least in part [12,13,14,32], and that DIF-1 would negatively modulate chemotaxis in shallow cAMP gradients via another receptor, DR-1C, whereas DIF-2 would positively modulate chemotaxis in shallow cAMP gradients via its receptor DR-2C. Note that DhkM, another receptor-type *Dictyostelium* histidine kinase, is involved in DIF-1-induced stalk cell differentiation (autophagic cell death) [36], and DhkM might be DR-1D [37]. (**B**) Chemical structures of the DIF-1-type molecules, DIF-1(3M) and TM-DIF-1, and scheme for their actions in inducing stalk cell differentiation and inhibiting chemotaxis via the putative DIF receptors. (**C**) Chemical structures of the DIF-2-type molecules, DIF-1A(+1) and TH-DIF-1, and scheme for their actions in inducing stalk cell differentiation and promoting chemotaxis via the putative DIF receptors. (**D**) Chemical structures of DIF-2, DIF-1A(+1), and TH-DIF-1, and scheme for their actions in promoting chemotaxis via the DhkC–RdeA–RegA pathway. DhkC might be DR-2C [20].

We have previously shown, by using DIF-1, DIF-2, and several amide derivatives of DIF-1 (Figure 1B), that DIF compounds differentially control stalk cell differentiation and chemotaxis. We hypothesized that DIFs have at least three receptors: DR-1D, responsible for stalk cell induction; DR-1C, a negative modulator of chemotaxis; and DR-2C, a positive modulator of chemotaxis (Figure 2BI) [11]. Although we have recently identified a DIF-binding protein, glutathione S-transferase 4 (Gst4), by utilizing DIF-conjugated resin and LC-MS/MS, Gst4 is unlikely to be a DIF receptor because in experiments with *gst^−^* cells DIF-1 induced stalk cell differentiation and DIF-1 and DIF-2 modulated chemotaxis [35]. The results of our experiments with the nine DIF derivatives (Figure 1C) supported our hypothesis about their mechanisms of action and the putative DIF receptors (Figure 6).

Several findings are helpful in identifying DR-1D and DR-1C. DIF-1 induces stalk cell differentiation, at least in part, via increases in cytoplasmic Ca^2+^ and H^+^ concentrations (Figure 6A) [12,13,14,32]. Furthermore, DIF-1 functions as a mitochondrial uncoupler both in *D. discoideum* and mammalian cells [38,39,40], but it was unknown if DIF-1 functions as an uncoupler during *D. discoideum* development. Recently, it was shown that DIF-1-BODIPY, a fluorescent derivative of DIF-1, that induced stalk cell differentiation and suppressed chemotaxis similar to DIF-1, was localized to mitochondria, suggesting that DIF-1 controls stalk cell differentiation and chemotaxis, at least in part, via mitochondria [21]. It should be noted that the mitochondrial uncouplers CCCP (carbonyl cyanide *m*-chlorophenylhydrazone) and DNP (dinitrophenol) induce partial stalk cell differentiation in HM44 cells in the presence of cAMP, and that CCCP and DNP, as well as DIF-1, suppress chemotaxis in the wild-type strain Ax2 in shallow cAMP gradients [21]. On the other hand, DIF-1 receptor(s) have been assumed to contain, or consist of, two distinct structures (receptors), S1 and S2 [36,41,42]; S1 is responsible for DIF-1-induced autophagic cell death (stalk cell differentiation) in wild-type cells and S2 is responsible for DIF-1-induced necrotic cell death in *atg1^−^* cells, an autophagy-deficient mutant [36]. Therefore, S1 may be DR-1D, or DR-1D might consist of two distinct motifs or subtypes, or it may involve two distinct co-factors that modulate DR-1D. DhkM, another receptor-type *Dictyostelium* histidine kinase, is involved in DIF-1-induced stalk cell differentiation [36]. Structural analysis using docking simulations and free energy evaluation showed that DhkM possesses a putative binding site for DIF-1 [37]. These results suggest that DhkM could be the receptor for DIF-1 (DR-1D) (Figure 6A); this could be verified by using DIF-1(3M) and DIF-1A(+1) in future studies (Figure 6B,C).

We hypothesized that DIF-2 interacts with the putative receptor DR-2C. Here, we showed that TH-DIF-1 and DIF-1A(+1) as well as DIF-2 negatively modulate chemotaxis, at least in part, via the DhkC–RdeA–RegA pathway (Figure 6D). DhkC is a receptor-type hybrid sensor kinase that carries domains homologous to the histidine kinase and receiver motifs of two-component phospho-relay signaling systems (Figure 2BII), and ammonia is thought to be an activator (ligand?) of DhkC in vivo [25,43,44]. However, because the receptors for cytokinins (plant hormones that are also small compounds) are histidine kinases and components of a two-component phospho-relay signaling system in plants [45,46] and also because the cytokinin discadenine is proposed to suppress spore formation through another *Dictyostelium* histidine kinase, DhkB, in *D. discoideum* [47,48], we suspect that DIF-2 is a ligand for DhkC, i.e., that DhkC might be DR-2C. This hypothesis could be verified by using DIF-2, TH-DIF-1, and DIF-1A(+1), in future studies (Figure 6D).

### 4.2. Utility of DIF Derivatives in the Study of Dictyostelium Development

Table 1 summarizes the chemotaxis-modulating and stalk cell-inducing activities of DIF compounds together with their chemical formulae, molecular weights, and cLogP values. The cLogP value indicates hydrophobicity, which would affect both membrane permeability of DIF compounds and ligand (DIF compound)–receptor interactions. Unfortunately, however, no significant correlation has been observed between the chemical structure of DIF compounds and either their chemotaxis modulation or their induction of stalk cell differentiation, nor between cLogP values and activity. However, we speculate that there might be some correlation between the hydrophobicity and activity of DIF compounds, and it is notable that DIF-1 and DIF-1(3M), which have the same chemical composition (C_13_H_16_C_l2_O_4_) and similar cLogP values (3.278 and 3.018, respectively) have nearly identical chemotaxis-modulating and differentiation-inducing activities. In addition, we would like to point out that DIF-2 and TH-DIF-1 have the same molecular weight of 293.14 and further that TH-DIF-1, a precursor of DIF-1 in vivo, possesses DIF-2-type activity in chemotaxis modulation in Ax2 cells but never induces stalk cell differentiation in HM1030 cells. These observations suggest that DIF receptors may not have strict structural specificity for ligands, similar to the aryl hydrocarbon receptor found in mammals, which can be activated by multiple lipophilic ligands [49,50].

The DIF derivatives tested here could be useful in future work. DIF-1(3M) can be used for analysis of the activity of DIF-1, including identification of DR-1D and DR-1C, since both DIF-1(3M) and DIF-1 induce stalk cell differentiation and negatively modulate chemotaxis (Figure 6A,B). TM-DIF-1 can be used for analysis of DR-1C because it negatively modulates chemotaxis without affecting stalk cell differentiation (Figure 6B). DIF-1A(+1) can be used for analysis of the activity of DIF-2 including the identification of DR-1D and DR-2C, since both DIF-1A(+1) and DIF-2 induce stalk cell differentiation and positively modulate chemotaxis (Figure 6A,C). TH-DIF-1 can be used for analysis of DR-2C since it positively modulates chemotaxis without affecting stalk cell differentiation (Figure 6C).

## 5. Conclusions

We examined the effects of nine derivatives of DIF-1 on chemotactic cell movement toward cAMP in *D. discoideum* and found that the DIF derivatives differentially affected chemotaxis and stalk cell differentiation. Our results suggest that the DIF-1 and DIF-2 have at least three receptors: one for stalk cell induction and two for chemotaxis modulation. Furthermore, the present results illuminate the DIF-signaling system and provide powerful tools for identifying DIF receptors in *D. discoideum*.

## Figures and Tables

**Table 1:** Effects of DIF compounds on chemotaxis and stalk cell formation.

		Chemotaxis Modulation in	Stalk Cell Induction * in		
Compounds	MW	Ax2 Cells	HM1030 (*dmtA^−^*) Cells	HM44 Cells	cLogP ***
		(100 nM DIFs) [Figure 3]	(20 nM DIFs) [Figure 5]	(2 nM DIFs) [26]	
DIF-1	307.17	🠳	+++++	+++++	3.278
DIF-2	293.14	🠱	+++	++++	2.749
DIF-3	272.73	ne [10]	±	±	2.895
TM-DIF-1	335.22	🠳	±	±	4.190
TH-DIF-1	293.14	🠱	± **	+++++ **	2.592
DIF-1(-2)	279.11	ne	nd	+	2.220
DIF-1(+1)	321.19	ne	nd	++++	3.807
DIF-1(+2)	335.22	ne	nd	++++	4.336
DIF-1(3M)	307.17	🠳	+++++	+++++	3.018
DIF-1(CP)	305.15	ne	nd	++++	2.634
I-DIF-1	490.08	ne	nd	+	4.008
Br-DIF-1	396.08	ne	nd	+++	3.678
		(10~100 nM DIFs) [11]	(10 nM DIFs) [11]	(0.5 nM DIFs) [19]	
DIF-1A(+1)	322.18	🠱	++++	++++	3.048
DIF-1A(+2)	336.21	ne	++++	+++	3.577
DIF-1A(+3)	350.24	🠳	±	±	4.106

Footnotes: MW, molecular weight; 🠳, suppression; 🠱, promotion; ne, no significant effect; nd, not determined. * Average stalk cell induction: ±, <10%; +, 10~20%; ++, 20~40%; +++, 40~60%; ++++, 60~80%; +++++, >80%. ** TH-DIF-1 is the immediate precursor of DIF-1 in DIF-1 synthesis in vivo. In HM44 cells, stalk cell formation was likely induced by DIF-1 derived from TH-DIF-1, but not in HM1030 cells. *** cLogP values differ slightly from those in our previous papers because we used a newer version of cLogP calculation software.

## Data Availability

Some or all data generated or analyzed during this study are included in this published article.
